# NMDA Suppresses Pancreatic ABCA1 Expression through the MEK/ERK/LXR Pathway in Pancreatic Beta Cells

**DOI:** 10.3390/nu16172865

**Published:** 2024-08-27

**Authors:** Takanobu Saheki, Hitomi Imachi, Kensaku Fukunaga, Seisuke Sato, Toshihiro Kobayashi, Takafumi Yoshimura, Nao Saheki, Koji Murao

**Affiliations:** Department of Endocrinology and Metabolism, Faculty of Medicine, Kagawa University, 1750-1, Ikenobe, Miki-cho, Kita-gun 761-0793, Japan; imachi.hitomi@kagawa-u.ac.jp (H.I.); fukunaga.kensaku@kagawa-u.ac.jp (K.F.); seisuke.310.med@gmail.com (S.S.); kobayashi.toshihiro@kagawa-u.ac.jp (T.K.); yoshimura.takafumi.j4@kagawa-u.ac.jp (T.Y.); yamajinao1121@yahoo.co.jp (N.S.); murao.koji@kagawa-u.ac.jp (K.M.)

**Keywords:** ABCA1, NMDA, LXR, pancreatic beta cells, type 2 diabetes, insulin secretion

## Abstract

Dysfunction or loss of pancreatic β cells can cause insulin deficiency and impaired glucose regulation, resulting in conditions like type 2 diabetes. The ATP-binding cassette transporter A1 (ABCA1) plays a key role in the reverse cholesterol transport system, and its decreased expression is associated with pancreatic β cell lipotoxicity, resulting in abnormal insulin synthesis and secretion. Increased glutamate release can cause glucotoxicity in β cells, though the detailed mechanisms remain unclear. This study investigated the effect of N-methyl-D-aspartic acid (NMDA) on ABCA1 expression in INS-1 cells and primary pancreatic islets to elucidate the signaling mechanisms that suppress insulin secretion. Using Western blotting, microscopy, and biochemical analyses, we found that NMDA activated the mitogen-activated protein kinase (MEK)-dependent pathway, suppressing ABCA1 protein and mRNA expression. The MEK-specific inhibitor PD98059 restored ABCA1 promoter activity, indicating the involvement of the extracellular signal-regulated kinase (MEK/ERK) pathway. Furthermore, we identified the liver X receptor (LXR) as an effector transcription factor in NMDA regulation of ABCA1 transcription. NMDA treatment increased cholesterol and triglyceride levels while decreasing insulin secretion, even under high-glucose conditions. These effects were abrogated by treatment with PD98059. This study reveals that NMDA suppresses ABCA1 expression via the MEK/ERK/LXR pathway, providing new insights into the pathological suppression of insulin secretion in pancreatic β cells and emphasizing the importance of investigating the role of NMDA in β cell dysfunction.

## 1. Introduction

Progressive β cell dysfunction and reduced β cell mass due to apoptosis are hallmarks of type 2 diabetes, but the exact causes of β cell dysfunction remain incompletely understood. Cholesterol accumulation leads to apoptosis in pancreatic β cells, and this phenomenon is known as β cell lipotoxicity [1]. Recently, this pathological condition has received significant attention because β cell lipotoxicity can lead to defective insulin synthesis and secretion. ABCA1 (ATP-binding cassette transporter A1) is a 254 kDa membrane protein that facilitates lipid transport across membranes, supporting HDL biosynthesis [2]. This process is crucial for initiating reverse cholesterol transport by promoting cholesterol efflux from the peripheral tissues. ABCA1 is extensively expressed across various tissues, including pancreatic β cells. In selective ABCA1 knockout mice, the intracellular cholesterol content in the pancreas is increased, and glucose-dependent insulin secretion (GSIS) is impaired [3], suggesting that cholesterol accumulation may lead to cell dysfunction. Current evidence suggests that ABCA1 plays a significant role in lipid metabolism and insulin secretion. Previously, we demonstrated that angiotensin II, TNF-α, and OxLDL suppress ABCA1 gene expression, induce lipotoxicity, and decrease GSIS in pancreatic β cells [4,5,6]. Conversely, GLP-1 promotes ABCA1 expression via the intracellular signaling pathway, ameliorating lipotoxicity and improving GSIS [7,8].

N-methyl-D-aspartate receptors (NMDARs) are a class of glutamate receptors found throughout the central nervous system. They are crucial for processes like memory and learning due to their interaction with glutamate neurotransmitters [9], while NMDA receptor inhibitors inhibit ATP-sensitive Kir6.1/Kir6.2 potassium channels and are used in the treatment of Alzheimer’s disease [10]. NMDARs are present in β cells, and elevated glutamate release leads to NMDAR-mediated β cell glucotoxicity and reduced insulin secretory capacity. However, the detailed mechanisms underlying glutamate-dependent inhibition of insulin secretion remain incompletely understood [11].

Using a rat pancreatic cell line, we investigated how NMDA contributes to cholesterol accumulation in β cells. This effect was mediated through an NMDA-dependent reduction of ABCA1 expression, leading to lipotoxicity, and suppression of insulin synthesis.

## 2. Materials and Methods

### 2.1. Cell Culture

INS-1 cells, originally developed by the Division of Biochimie Tabl Clinique and provided by C. B. Wollheim from Geneva, Switzerland, were utilized in this study. These cells, derived from rat insulinoma, were passaged every 7 days, and experiments were conducted using cells between passages 15 and 35. The cells were cultured in RPMI 1640 medium (Sigma, Tokyo, Japan) enriched with 10% heat-inactivated fetal bovine serum (FBS; Dainippon Pharmaceutical, Tokyo, Japan), 11.2 mmol/L glucose, 50 μM β-mercaptoethanol, 100 µg/mL streptomycin, and 100 U/mL penicillin. They were incubated in a humidified incubator with 5% CO_2_ at 37 °C. Cells reaching 80% confluence were washed twice and starved for 6 h in RPMI 1640 medium containing 0.5% FBS. Post-starvation, the cells were treated with varying concentrations of NMDA for 24 h or at different time points. Before NMDA treatment, the NMDA inhibitor MK801 was applied for 30 min randomly.

### 2.2. Pancreatic Islet Culture

Rat pancreatic islets obtained from Cosmo Bio Co., Ltd. (Tokyo, Japan) were cultured in RPMI 1640 medium supplemented with 10% heat-inactivated FBS, 100 U/mL penicillin, and 0.1 mg/mL streptomycin and incubated in 5% CO_2_ at 37 °C. After 6 h of fasting, the islets were treated with 1 mM NMDA for 24 h before protein extraction. Harvested islets were used for experiments over a span of 7 days.

### 2.3. Western Blot Analysis

Proteins (15–30 μg) were separated using SDS-PAGE with a 7.5% gel and subsequently transferred to membranes for immunoblotting. Following an overnight blocking step at 4 °C using 7.5% skim milk, the membranes were treated with primary antibodies targeting ABCA1 (254 kDa), the NMDA receptor (NMDAR, 120 kDa), the liver X receptor (LXR, 63 kDa), or mitogen-activated protein kinase (MEK, 45 kDa), phospho-MEK, ERK 1/2(44/42 kDa), and phospho-ERK 1/2 (Cell Signal Technology, Tokyo, Japan) at 4 °C overnight. In addition, glyceraldehyde 3-phosphate dehydrogenase (GAPDH, 36 kDa) antibody (Biomol Research, Plymouth Meeting, PA, USA) was applied for 1 h at room temperature [4]. After the primary antibody incubation, the membranes were washed three times with PBS-T for 10 min each and then incubated with horseradish peroxidase-conjugated secondary antibodies (mouse or rabbit, DakoCytomation, diluted 1:2000) for 1 h at room temperature. The membranes were washed three more times with PBS-T for 10 min each. Antigen–antibody complexes were detected using an ECL detection system (GE Healthcare, Tokyo, Japan). Antibodies were validated based on the published literature, with the specificity and sensitivity of the primary antibodies confirmed using the following references: ABCA1 [12], NMDA receptor [13], liver X receptor, MEK, phospho-MEK, ERK 1/2, phospho-ERK 1/2 [6], and GAPDH [4] antibodies. Additionally, secondary antibodies were validated for probe conjugation and specificity according to the protocols provided by the manufacturer.

### 2.4. Real-Time Polymerase Chain Reaction (PCR)

PCR was performed using 20 µL of LightCycler (Bio-Rad, Hercules, CA, USA), including 10 µL SYBR Green PCR Master Mix, 0.5 µM of each primer (forward: 5′-CCCGGCGAGTAGAAAGG-3′ and reverse: 5′-AGGGCGATGCAAACAAAGAC-3′), and a 1 µL DNA template. The cycling conditions were as follows: initial denaturation at 95 °C for 3 min, followed by 40 cycles of denaturation at 95 °C for 15 s, annealing at 60 °C for 30 s, and extension at 72 °C for 30 s. A melting curve analysis was conducted by gradually increasing the temperature from 65 °C to 95 °C to confirm specific amplification. Water was used as a negative control to check for contamination. Known concentrations of DNA were prepared as standards, and their crossing points were used to generate a standard curve using LightCycler 2.1. software. The regression curve of crossing points versus DNA concentration was analyzed to determine the gene expression levels [14]. GAPDH served as a housekeeping gene for normalization.

### 2.5. Luciferase Reporter Gene Assay

INS-1 cells were transfected with purified pABCA1-LUC using Lipofectamine 2000 (Life Technologies, Gaithersburg, MD, USA). After transfection, they were incubated with 10 μM PD98095(2- (2-Amino-3-methoxyphenyl)-4H-1-benzopyran-4-one), H89(N-[2-(p-Bromocin-namylamino)ethyl]-5-isoquinolinesulfonamide2HCl), LY294002(2-(4-Morpholinyl)-8-phenyl-4H-1-benzopyran-4-one), and SB203580(4-(4-fluorophenyl)-2-(4-methylsulfinyl-phenyl)-5-(4-pyridyl)-imidazole) and maintained in medium containing NMDA for 24 h. They were then individually treated with MEK, protein kinase A (PKA), phosphatidylinositol 3 kinase (PI3K), or p38 mitogen-activated protein kinase (p38 MAPK) for 30 min to induce upregulation of the respective signaling pathways [4]. After harvesting the transfected cells, the promoter activity of ABCA1 was assessed within a portion of the cytoplasmic preparation. For the luciferase assay, 40 μL aliquots were used, following the instructions provided by the manufacturer (ToyoInk, Tokyo, Japan).

### 2.6. Glucose-Stimulated Insulin Secretion

INS-1 and islet cells were incubated with 1 mM NMDA or 10 µM LXR ligand (22(R)-hydroxycholesterol) in culture medium for 24 h, following a 6 h starvation period [6,15]. The culture medium was then replaced with Krebs-Ringer bicarbonate (KRB) buffer containing 120 mM NaCl, 1.1 mM MgCl_2_, 2.5 mM CaCl_2_, 5 mM KCl, 25 mM NaHCO_3_, and 0.1% bovine serum albumin (pH 7.4) for 1 h. The cells were treated with KRB buffer containing 3.3 mM glucose for 1 h, followed by continuous treatment with 16.7 mM glucose buffer for 1 h. After 1 h of incubation, the supernatant and cell proteins were collected and used for insulin measurement with an ELISA kit (Shibayagi, Japan).

### 2.7. Cholesterol and Triglyceride Content Assay

The cells subjected to different treatments were washed twice with PBS and resuspended in 800 µL PBS. Of this suspension, 450 µL was treated with Triton X-100 for cholesterol and triglyceride analysis using the ARCHITECT c8000 (Abbott Laboratories, Abbott, IL, USA), while the remaining 350 µL was used for cell counting. The cholesterol and triglyceride concentration per cell was determined by calculating the ratio of cholesterol content (µg/mL) to the number of cells (cells/mL). After washing with PBS (pH 7.2), the cells were lysed in HEPES buffer containing 1% Triton X-100. The cholesterol and triglyceride levels were measured using a colorimetric assay on an ARCHITECT c8000 analyzer with standard reagents for cholesterol [16].

### 2.8. Oil Red O Stain

The INS-1 cells were plated on coverslips, treated with NMDA and MK801, and fixed with 4% paraformaldehyde for 30 min at room temperature. After three washes with PBS, the cells were stained with Oil Red O for 15 min. The cells were then rinsed three times with PBS, and the nuclei were stained with hematoxylin. After additional PBS washes, the samples were mounted on coverslips. Cell images were captured using an Olympus BX-51/DP-72 microscope (Olympus Corporation, Tokyo, Japan). For quantification of the Oil Red O staining, the cells were washed with 60% 2-propanol to remove Oil Red O from the plate and quantified. Then, 4% Triton X-100 in 2-propanol was added and allowed to stand for a few minutes. After confirming that the cells had turned white, the Oil Red O extract was placed in a tube, and the absorbances of the Oil Red O standard and the extract were then measured to determine the concentration.

### 2.9. Statistical Analysis

Data are presented as mean ± standard error of the mean (s.e.m.). Statistical significance was assessed using one-way ANOVA, with *p* < 0.05 considered statistically significant. All experiments were performed in triplicate or more.

## 3. Results

### 3.1. NMDA Decreased ABCA1 Expression in INS-1 Cells and Islets Cells

**Figure 1:**
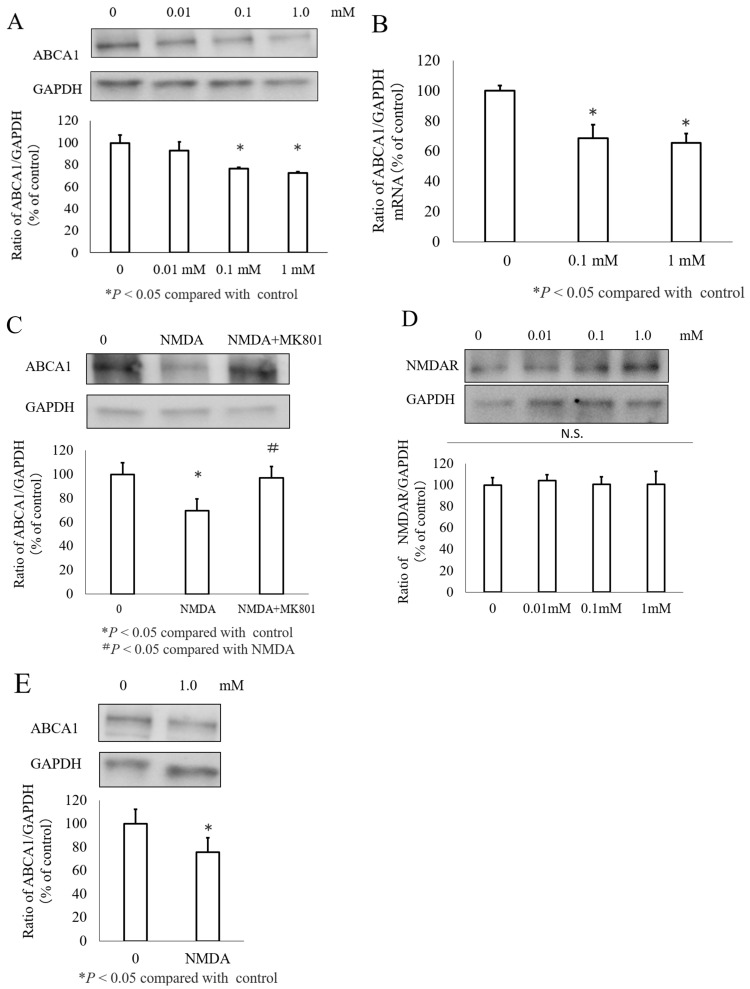
Impact of varying concentrations of NMDA on ABCA1 expression in INS-1 and rat islet cells. (**A**) Abundance of ABCA1 protein after treatment with NMDA at concentrations of 0, 0.01, 0.1, and 1 mM. (**B**) ABCA1 mRNA levels after exposure to NMDA at concentrations of 0, 0.01, 0.1, and 1 mM. (**C**) ABCA1 expression in INS-1 cells treated with NMDA or MK801, expressed as a percentage of the control, with ABCA1 levels normalized to GAPDH. Data are presented as mean ± SEM from three independent experiments (* *p* < 0.05 compared with the control; # *p* < 0.05 compared to NMDA). (**D**) Expression of NMDA receptor. (**E**) ABCA1 protein levels in rat islets following treatment with 0 or 1 mM NMDA.

Total protein was first extracted from the INS-1 cells treated with varying concentrations of NMDA (0–1000 µM), and ABCA1 expression was assessed using Western blotting. The results indicated a dose-dependent reduction in ABCA1 expression in the INS-1 cells following NMDA treatment (Figure 1A). This was confirmed using real-time PCR (Figure 1B). Because NMDA efficiently reduced ABCA1 expression at a concentration of 1 mM, this concentration was used in subsequent experiments. We then used an N-methyl-D-aspartate (NMDA) inhibitor (MK801) to examine whether NMDA-induced ABCA1 inhibition could be reversed. Our findings indicate that MK801 treatment successfully abolished the effects of NMDA and restored ABCA1 expression (Figure 1C). To confirm the NMDAR expression, the cells were treated with different concentrations of NMDA, and Western blotting confirmed the decreased expression of total NMDARs (Figure 1D). We also confirmed the effect of 1 mM NMDA on the rat pancreatic islets by observing a decrease in protein levels following NMDA treatment (Figure 1E).

### 3.2. NMDA Decreased ABCA1 Transcription in INS-1 Cells via the MEK/ERK Signaling Pathway

**Figure 2:**
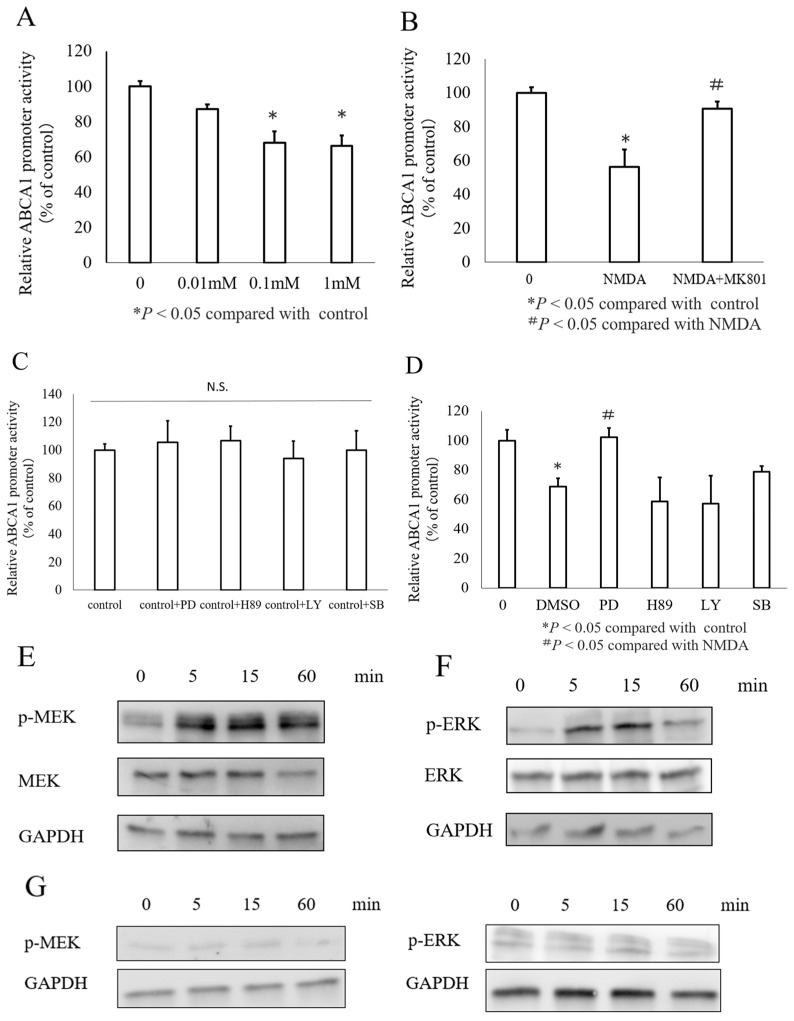
Involvement of NMDA in ABCA1 promoter activity in INS-1 cells. (**A**) ABCA1 promoter activity in INS-1 cells treated with various concentrations of NMDA (0, 0.01, 0.1, and 1 mM). (**B**) ABCA1 promoter activity in INS-1 cells treated with NMDA and MK801. (**C**) Various effects on ABCA1 promoter activity in the absence of NMDA after treatment with inhibitors of different signaling pathway components (PD for MEK, H-89 for PKA, LY for PI3K, and SB for p38 MAPK). The percentage of promoter activity was compared to that of the control cells. N.S., not significant. (**D**) Varying effects on ABCA1 promoter activity following treatment with 1 mM NMDA or inhibitors of different signaling pathways. (**E**–**G**) Phosphorylation of MEK at Ser 217/221 and ERK at Thr 202/Tyr 204 in INS-1 cells treated with NMDA (**E**,**F**) and without NMDA (**G**) (* *p* < 0.05 compared to control; # *p* < 0.05 compared to NMDA).

To examine whether NMDA affects *ABCA1* transcription, INS-1 cells were transfected with a luciferase reporter plasmid containing the promoter region of *ABCA1* and treated with NMDA at concentrations of 0, 0.01, 0.1, and 1 mM. As expected, NMDA caused a dose-dependent decrease in *ABCA1* promoter activity (Figure 2A), and this effect was abrogated by treatment with MK801 (Figure 2B). Next, we examined the signal transduction pathways involved in mediating the effects of NMDA on *ABCA1* expression. We inhibited the MEK, PKA, PI3K, and p38MAPK signaling pathways using inhibitors PD (10 μM), H89 (1 μM), LY (10 μM), and SB (1 μM), respectively for each component. Initially, the effect of the inhibitor on the control group was not significant (Figure 2C). Next, we tested the effects of NMDA and its inhibitors, revealing that only PD effectively blocked NMDA’s inhibitory impact on *ABCA1* promoter activity (Figure 2D). This finding suggests that NMDA modulates *ABCA1* expression through the MEK signaling pathway in INS-1 cells. To further investigate, the INS-1 cells were exposed to NMDA for varying durations (0–60 min), and the activation of the MEK/ERK signaling pathway was assessed. Western blotting showed that Ser217/221-phosphorylated MEK levels peaked 5 min after NMDA treatment, persisted until 15 min, and then returned to baseline at 60 min (Figure 2E). Similarly, Thr202/Tyr204-phosphorylated ERK 1/2 was activated between 5 and 15 min (Figure 2F). Next, we used the INS-1 cells without NMDA treatment and conducted experiments at various time points (0–60 min). No changes in phosphorylation were observed (Figure 2G).

### 3.3. Effects of NMDA on Lipid Accumulation in INS-1 Cells

**Figure 3:**
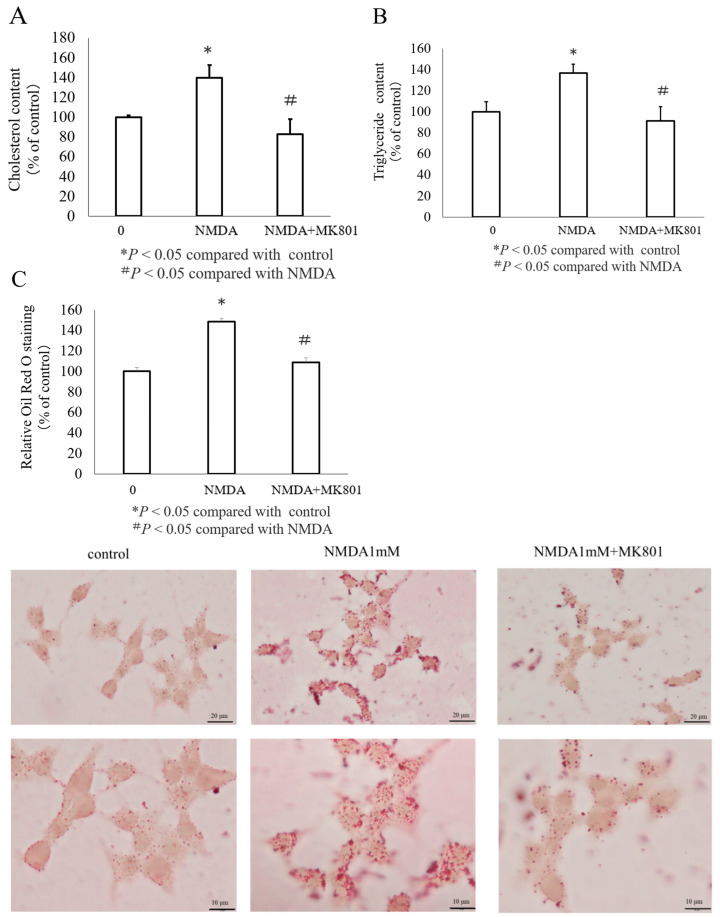
Impact of NMDA on the accumulation of cholesterol and triglycerides in INS-1 cells. Cholesterol content (**A**), triglyceride content (**B**), and Oil Red O staining (**C**) in INS-1 cells with varying treatments. (**A**,**B**) The percentage of cholesterol and triglyceride content per cell relative to the control is presented as mean ± SEM from three independent experiments (* *p* < 0.05 compared with the control; # *p* < 0.05 compared to NMDA). (**C**) Oil Red O staining results are displayed. The top image was taken at a low magnification (scale bar = 20 μm), and the bottom image was taken at a higher magnification (scale bar = 10 μm).

We conducted experiments to understand how NMDA influences lipid accumulation in INS-1 cells. As shown in Figure 3A, the cholesterol content increased to 139 ± 13.2% compared to that of the control after NMDA treatment. Further, the triglyceride content was significantly increased by NMDA (Figure 3B). Intracellular lipid accumulation was assessed using Oil Red O staining. Based on our imaging analysis, the number of fat droplets became larger and increased after NMDA administration (Figure 3C). This was confirmed by quantifying the Oil Red O staining. Notably, the effects of NMDA on the cholesterol content and lipid droplet size and number were reversed by treatment with MK801 (Figure 3A–C). Overall, these results indicate that NMDA increases lipid accumulation in INS-1 cells.

### 3.4. GSIS Is Reduced by NMDA

**Figure 4:**
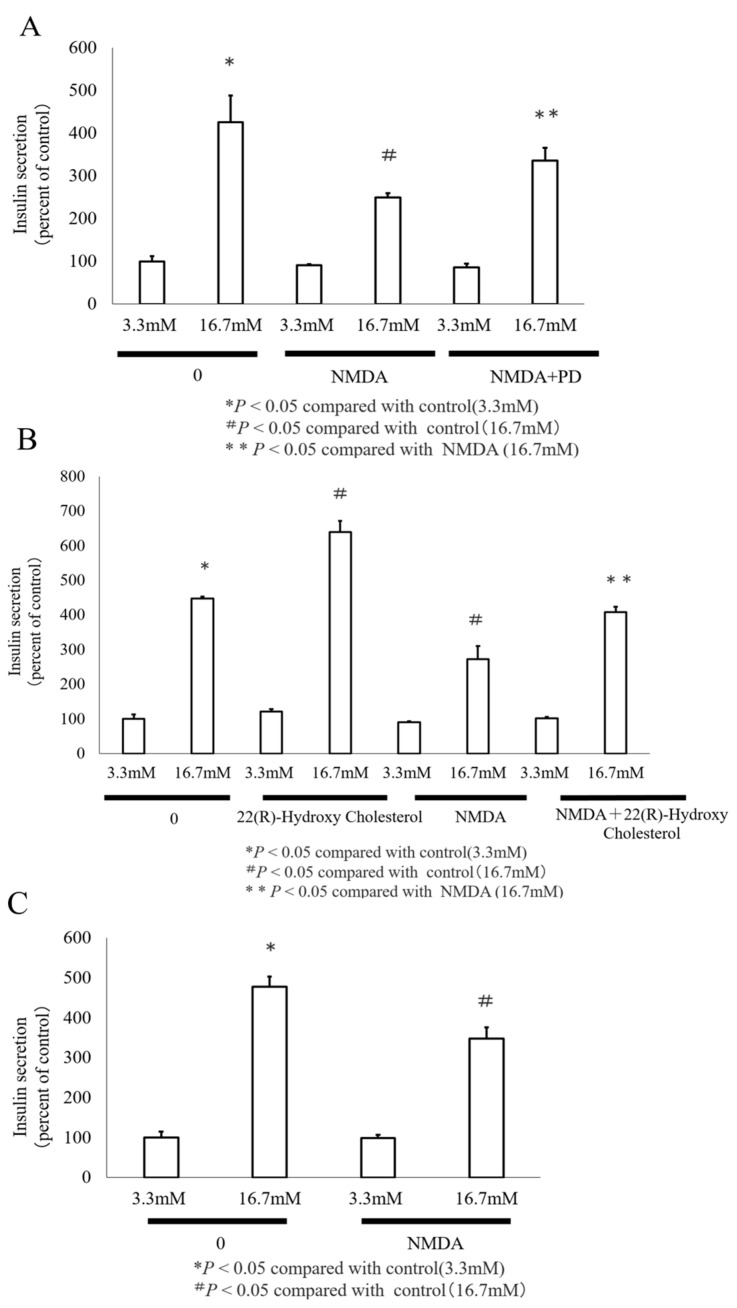
Effect of NMDA on insulin secretion in INS-1 cells. (**A**) Insulin secretion was confirmed by comparing the effects of low-glucose (3.3 mM) and high-glucose (16.7 mM) treatments after a 24 h incubation with NMDA at 1 mM concentration and NMDA + the MEK inhibitor PD (NMDA + PD). (**B**) Insulin secretion was confirmed using LXR ligand and NMDA. (**C**) GSIS was measured in primary pancreatic islets from rats treated with 1 mM NMDA. Insulin secretion was confirmed under low- to high-glucose conditions. * *p* < 0.05 compared with control (3.3 mM); # *p* < 0.05 compared with control 16.7 mM); ** *p* < 0.05 compared with NMDA (16.7 mM).

Lipid accumulation in pancreatic β cells induced a decrease of GSIS. Next, we assessed the impact of NMDA on GSIS. As shown in Figure 4A, high-glucose treatment (16.7 mM) in the control group resulted in a significant increase in insulin secretion to 426 ± 62.5% compared to low-glucose treatment. In contrast, the INS-1 cells treated with 1 mM NMDA exhibited a reduced insulin secretion level of 275 ± 11.1%, which was significantly lower than the control under high-glucose conditions. PD improved insulin secretory response to high-glucose stimulation. Insulin secretion was also increased when LXR ligand was used. LXR ligand also restored insulin secretion suppressed by NMDA (Figure 4B). These findings indicate that NMDA-induced lipotoxicity reduces insulin secretion. Collectively, the results highlight that NMDA reduces GSIS through the MEK/ERK signaling pathway. Similar effects on GSIS were obtained in the rat pancreatic islets (Figure 4C).

### 3.5. The Transcription Factor LXR Mediated NMDA Induced ABCA1 Inhibition

**Figure 5:**
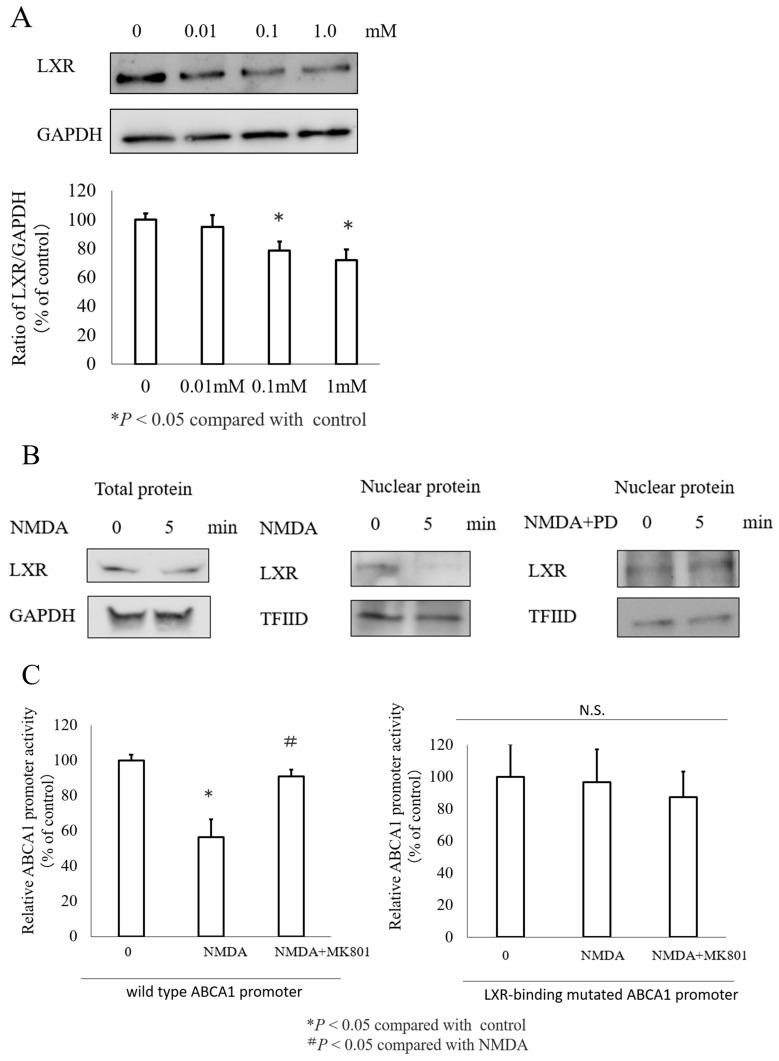
Role of the transcription factor LXR in NMDA-suppressed ABCA1 expression. (**A**) Total LXR protein levels in INS-1 cells following a 24 h treatment with NMDA at concentrations of 0, 0.01, 0.1, and 1 mM. (**B**) Total and nuclear LXR protein levels in INS-1 cells treated with NMDA or NMDA and PD for 5 min. (**C**) Introduction of a site-directed mutation in the LXR binding site altered the base pair sequence from GATAGT to AATAGG in the ABCA1 promoter region, inhibiting binding of LXR. N.S., not significant.

Next, we analyzed the transcription factors that promote NMDA-mediated inhibition of *ABCA1* gene expression. Many transcription factors bind to *ABCA1* promoter lesions. Our previous research demonstrated that LXR binds to the *ABCA1* promoter region and that co-expression of LXR with RXR markedly increases ABCA1 promoter activity [17]. To investigate the impact of NMDA on this process, we examined the role of LXR in NMDA-induced suppression of *ABCA1*. Figure 5A shows that NMDA caused a dose-dependent reduction in LXR expression. The expression of LXR was also suppressed in the cell nuclear extracts 5 min after treatment with NMDA. Furthermore, when MEK inhibitors were used to confirm the expression of LXR, the reduction of LXR expression in the cell nucleus extracts was canceled (Figure 5B). Finally, we introduced a mutation in the *ABCA1* promoter’s binding site for LXR to confirm the role of LXR in mediating the inhibitory effect of NMDA on *ABCA1* expression. Figure 5C shows that NMDA inhibited the activity of the wild type *ABCA1* promoter but did not affect the activity of the mutated *ABCA1* promoter. These results indicate that LXR is essential for NMDA-mediated regulation of *ABCA1* expression.

## 4. Discussion

NMDAR expression is not only present in central and peripheral glial cells but is also found in various non-neuronal tissues, such as endothelial cells, the kidney, bone, and pancreas [18]. Despite this, the impact of glutamate on insulin secretion continues to be a topic of debate. Some reports have suggested that NMDA does not affect insulin secretion in beta cells [18], whereas others have reported an increase in insulin secretion following short-term treatment with NMDA [19]. Furthermore, there are findings suggesting that NMDAR inhibitors increase insulin secretion [20]. Prolonged exposure to NMDA is associated with a reduction in GSIS in β cells [11]. NMDAR inhibition in mice enhances GSIS [21]. However, continuous administration of NMDA induces apoptotic β cell death, mediated by inflammatory cytokines and endoplasmic reticulum stress, leading to impaired insulin secretion [22]. In addition, NMDA causes Ca^2+^ influx, activates AMP-activated protein kinase, and increases transport of Kv2.1 and KATP channels to the plasma membrane, resulting in membrane hyperpolarization [23]. Elevated glutamate levels have been observed in patients with diabetes and obesity [24]. In addition, prolonged NMDAR activation in patients with diabetes and mice is believed to cause β cell dysfunction and glucotoxicity [11,25,26]. Therefore, NMDAR-mediated NMDA actions are thought to contribute to β cell dysfunction, resulting in decreased insulin secretion. However, the effects on intracellular signaling remain unclear. In this study, we identified NMDA-induced lipotoxicity using a pancreatic β cell model system. INS-1 cells were employed for these experiments, but future studies should use human islets or other cell lines to validate these results. Due to the challenges of obtaining human β cell lines or isolated human islets, the effects of NMDA were confirmed using primary cultured rat islets, in which NMDA reduced ABCA1 expression. This study explored the relationship between ABCA1 and NMDA in pancreatic β cells. It is important to highlight that ABCA1 plays a crucial role in lipid metabolism and exerts protective effects against arteriosclerosis [27]. Furthermore, concerning the relationship between ABCA1 and NMDAR, studies have demonstrated that NMDAR activation in macrophages triggers calpain-mediated degradation of the ABCA1 protein, resulting in reduced surface expression of ABCA1, accelerated lipid accumulation, disrupted lipid metabolism, and foam cell formation [28].

Given the lack of reports on the effects of NMDA in pancreatic β cells concerning ABCA1 expression and lipotoxicity, we investigated the possibility that NMDA and ABCA1 may be involved in lipotoxicity in these cells. We demonstrated that NMDA decreases insulin secretion even under high-glucose conditions. NMDA promotes lipid accumulation, leading to pancreatic β cell dysfunction and lipotoxicity. This study did not examine the direct effects of NMDA on insulin secretion in INS-1 cells. Additionally, the effect of short-term NMDA application during GSIS remains unexplored. These issues should be considered in future studies.

We confirmed that NMDA decreases GSIS levels via the MEK/ERK signaling pathway. We assume that the GSIS results are attributable to changes in intracellular Ca^2+^ concentration. However, this study did not confirm this, so we suggest that it should be a subject for future research.

An objective was to delineate the signaling pathways involved in NMDA-regulated *ABCA1* gene expression. NMDA suppressed *ABCA1* promoter activity, which was blocked by PD98059, an MEK-specific inhibitor. This suggests that NMDA regulates *ABCA1* expression through the MEK signaling pathway in INS-1 cells. MEK is an MAP upstream of ERK, which phosphorylates and upregulates ERK [29]. In the present study, NMDA treatment activated the Ser217/221 site of MEK and led to subsequent activation of the Thr202/Tyr204 site of ERK. These results reveal that *ABCA1* expression in INS-1 cells is controlled by the MEK/ERK pathway, which is also responsible for mediating the effects of NMDA on this transporter gene. Furthermore, we examined the downstream transcription factors involved in NMDA-dependent regulation of *ABCA1* expression. Our previous study indicated that the transcription factor LXR may be an essential regulator of *ABCA1* promoter activity. LXR is activated by oxysterol ligands and forms a heterodimer with RXR. This complex binds to the LXR response element in the gene promoter and upregulates *ABCA1* expression [30]. LXR regulates cholesterol levels by promoting cholesterol efflux and protecting cells from lipotoxic damage [31]. Activation of the DR4 site of the ABCA1 promoter by LXR upregulates ABCA1, which is important for cholesterol efflux [15]. Our study found that LXR is necessary for effectively suppressing ABCA1 expression. The LXR ligand affected GSIS in INS-1 cells, consistent with previously reported findings [32] and restoring insulin secretion inhibited by NMDA.

The global prevalence of diabetes is increasing, underscoring the need for continued advancement in diabetes research. Various antidiabetic drugs are used to manage hyperglycemia in patients with diabetes mellitus. The connection between KATP channels associated with sulfonylureas and NMDA has been previously recognized. The KATP channel comprises a potassium channel subunit Kir6.x and sulfonylurea receptor (SUR) subunits. Inhibition of NMDA receptors inactivates KATP channels, resulting in improved blood glucose levels in type 2 diabetic mice [20]. However, memantine does not affect KATP channels in pancreatic β cells [18]. The effect of NMDA on Kir6.x and SUR channels remains unexplored and warrants further investigation. To address this gap, our future research will examine how NMDA influences the Kir6.x and SUR channels. It is crucial to develop treatments that enhance and restore pancreatic islet function without causing excessive insulin secretion or contributing to obesity. Various treatments have been explored to improve insulin secretion, a key factor in diabetes management. For instance, stimulating only the vagus nerve connected to the pancreas elevates blood insulin levels and increases the number of β cells more than two-fold [33]. In addition, therapy using pancreatic islet cells derived from stem cells has been researched [34]. A small clinical trial in which dextromethorphan (DXM), an NMDAR antagonist, was orally administered to patients with diabetes showed improved glucose tolerance and increased insulin secretion [35]. Oral administration of the NMDAR inhibitors DXM and amantadine increased insulin concentrations during a 75 g oral glucose tolerance test (OGTT) [20]. Furthermore, the combination of DXM and sitagliptin significantly increased initial insulin secretion and exerted hypoglycemic effects compared with sitagliptin alone in a 75 g OGTT [35]. These findings suggest that NMDAR antagonists may promote increased insulin secretion and β cell protection, offering potential as antidiabetic agents. However, long-term clinical trials have not yet been conducted, and side effects, such as euphoria and hallucinations, are commonly reported [36], necessitating caution.

Therefore, the clinical use of NMDA antagonists remains limited, highlighting the need for further studies to address these issues. Our current work revealed a signaling pathway through which NMDA leads to decreased insulin secretion associated with lipotoxicity in pancreatic β cells. These findings further support that NMDARs are novel candidate targets for therapeutic approaches to ameliorate insulin hyposecretion. Treatment strategies may be further optimized by considering combination therapies that include NMDAR antagonists. These advances could lead to more effective and targeted therapies for diabetes, potentially improving patient outcomes and offering new avenues for managing this chronic disease.

## 5. Conclusions

In conclusion, NMDA suppresses pancreatic ABCA1 expression through the MEK/ERK/LXR pathway, resulting in cholesterol accumulation and decreased glucose-dependent insulin secretion. Although the mechanism by which NMDA suppresses GSIS in pancreatic β cells remains unclear, NMDARs may represent new therapeutic agents targeted against diabetes. However, further clinical and in vivo studies are required to confirm the generalizability of our findings.

## Data Availability

The data supporting the results of this study can be obtained from the corresponding author upon request. The data are not publicly available due to ethical reasons.
